# Changes in microglia during drug treatment of stroke

**DOI:** 10.1002/ibra.12037

**Published:** 2022-05-22

**Authors:** Ling‐Jing Zhang, Hong‐Su Zhou, Shi‐Ya Wang, Yi‐Huan Guan, Chao Zhang, De‐Rong Fang

**Affiliations:** ^1^ Department of Anesthesia Zunyi Medical University Zunyi Guizhou China; ^2^ Department of Anesthesia Graduate School of Zunyi Medical University Zunyi Guizhou China; ^3^ Department of Anesthesiology Affiliated Hospital of Zunyi Medical University Zunyi Guizhou China; ^4^ Department of Family Planning Affiliated Hospital of Zunyi Medical University Zunyi Guizhou China

**Keywords:** medicine, microglia, stroke

## Abstract

Microglia are the main immune cells in the brain and the first defense barrier of the nervous system. Microglia play a complex role in the process of stroke. A growing number of studies focus on the mechanism of action of drugs functions and how to regulate microglia. Therefore, we talk about the pathophysiological mechanisms of stroke and elaborate on the microglia signaling pathways of drug action in stroke models and how these drugs play a role in stroke treatment in this review. Understanding how drugs modulate proinflammatory and anti‐inflammatory responses of microglia may be critical to implementing therapeutic strategies using immune interventions in stroke.

## Graphical abstract

A growing number of studies confirm that microglia play an essential role in the occurrence and development of stroke. During the long research years, researchers have discovered that the majority of drugs used to treat stroke alleviate damage to the central nervous system by modulating signaling pathways within microglia to treat stroke.

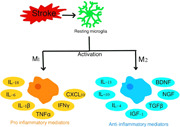

## 1 INTRODUCTION

**Figure 1:**
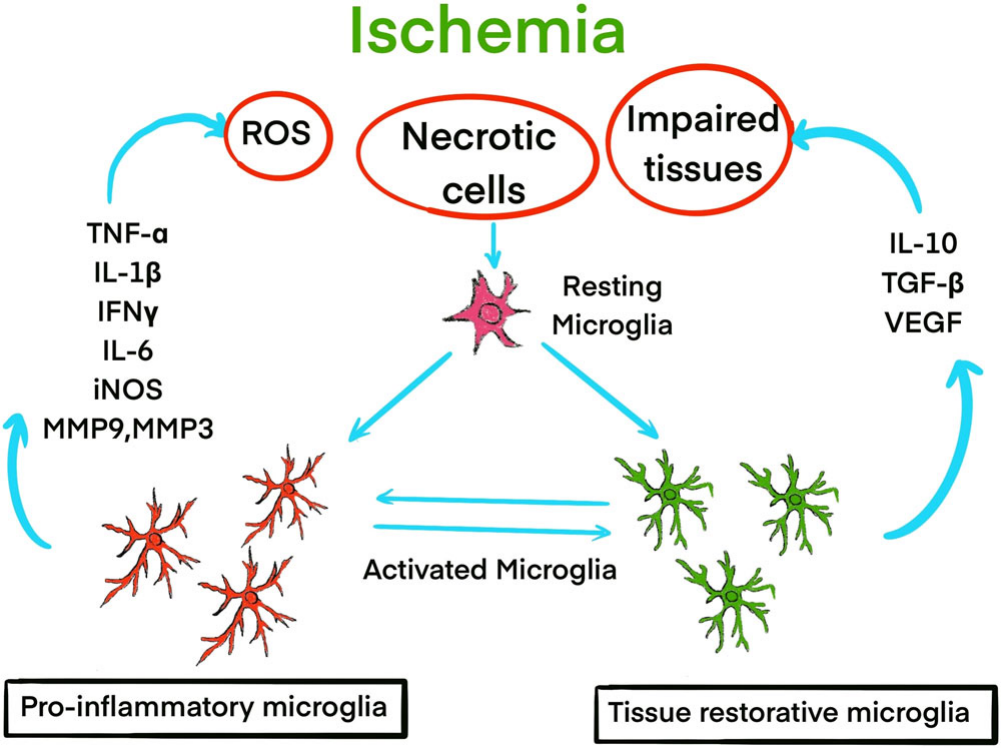
In ischemic stroke, resting microglia are activated and polarized into functionally distinct phenotypes that range between two extremes. Classical microglia produce proinflammatory mediators, including tumor necrosis factor‐α (TNF‐α), interleukin‐1 beta (IL‐1b), interferon‐c (IFNc), interleukin‐6 (IL‐6), inducible nitric oxide synthase (iNOS), and proteolytic enzymes (MMP9, MMP3), identified as proinflammatory. Alternative microglia are characterized by the production of IL‐10, transforming growth factor b (TGFb), and vascular endothelial growth factor (VEGF), which are proangiogenic and anti‐inflammatory. Alternative microglia are associated with tissue repair and remodeling, immunity against parasites, and growth stimulation. [Color figure can be viewed at wileyonlinelibrary.com]

Most strokes are caused by sudden insufficient blood supply, usually because of a blocked artery, and a rarer type of ischemic stroke is an infarct of a vein due to occlusion of a vein or sinus in the brain. According to regional epidemiology, the remaining 10%–40% of strokes are hemorrhages, the result of ruptured cerebral arteries.^1^ Stroke causes the death of 5.5 million people annually and is the secondary cause of death in the world. Globally, more than 80 million people have been reported to have survived a stroke, with one in four adults experiencing a stroke in their lifetime.^2^ In 2016, the incidence of ischemic stroke was 9.5 million.^3^ Up to now, the clinical treatment of stroke mainly includes drug therapy (Table 1), neuroprotective therapy, surgery, gene therapy, and so forth. A growing number of studies have shown that in microglia‐like peripheral macrophages, there are two completely different activation states to function. Interferon‐γ (IFN‐γ), interleukin‐1β (IL‐1β), IL‐12, and IL‐6, as well as lipopolysaccharide, induce classically activated microglia (M1) contributing to the generation and releasing proinflammatory cytokines,^4^, ^5^ which are involved in inflammation (Figure 1).

**Table 1:** The role of drugs in the treatment of cerebral ischemia

Medicine	Function	References
*Gardenia jasminoides* J. Ellis (GJ‐4)	GJ‐4 inhibits the Janus kinase 2 (JAK2)/signal transducer and activator of the transcription 1 (STAT1) pathway to suppress microglia activation and decrease the inflammatory protein expression.	[6]
Melatonin	Shifting the microglia phenotype from proinflammatory to anti‐inflammatory polarity in a STAT3‐dependent manner	[7]
Wnt‐3a	Improving the microglia/macrophage and astrocyte toxicity response in ischemic brain injury	[8]
Kellerin from Ferula sinkiangensis	Kellerin from Ferula sinkiangensis inhibits microglia activation and reduces proinflammatory cytokine levels and inhibits NF‐κB signaling pathway, reduces ROS production and NADPH oxidase activity	[9]
Pterostilbene	Inhibition of microglia ROS/NF‐κB‐mediated inflammatory pathways	[10]
Tanshinol borneol ester (DBZ)	DBZ inhibits NF‐κB activity, enhances Nrf2 nuclear accumulation and transcriptional activity, and alters M1/M2 polarization	[11]
Stepharine	Inhibition of TLR4/NF‐κB pathway and microglia hyperactivation	[12]
TWS119	Improving the neuroinflammatory microenvironment by modulating the shift of microglia toward an anti‐inflammatory phenotype	[13]
6‐Gingerol	Inhibition of microglia‐mediated neuroinflammatory responses through downregulation of the Akt‐mTOR‐STAT3 pathway	[14]
Cottonseed oil	Inhibition of TLR4/NF‐κB pathway and reduction of A1 phenotypic neurotoxic astrocyte activation	[15]
β‐caryophyllene	Suppressing microglia activation and regulating their polarization through the TLR4 pathway to exert its anti‐inflammatory effects	[16]
Scutellarin	Anti‐inflammatory effects on AM/BM via MAPKs pathway	[17]
PAP‐1 (Kv1.3 channel blocker)	Reducing microglia NLRP3 inflammatory vesicle activation by shifting the microglia phenotypic response from M1 to M2	[18]
8e	Regulation of superoxide formation in activated microglia via the PI3Kγ/AKT/NOX2 signaling pathway, which, in turn, prevents neuronal death in adjacent neurons	[19]
Calycosin	Enhancing endogenous BDNF production and microglial activation	[20]
Eerdun Wurile	Strengthening the expression of Igf1 and Igf2 in neurons and microglia	[21]
Minocycline	Promoting microglia M2 polarization and inhibiting M1 polarization via the STAT1/STAT6 pathway	[22]
Xueshuantong injection (lyophilized) combined with salvianolate lyophilized injection	Inhibition of oxidative stress and the Nrf‐2/Keap1 pathway	[23]
Berberine (BBR)	Promoting functional recovery and angiogenesis after MCAO through AMPK‐dependent microglia M2 polarization	[24]
Matrix metalloproteinases (MMPs)	Promoting activation and migration of astrocytes and microglia after cerebral ischemia	[25]
XQ‐1H	Regulation of pro/anti‐inflammatory microglia polarization balance via the PPARγ pathway	[26]
Celastrol	Decreasing OGD‐induced inflammatory cytokine expression through IL‐33/ST2 axis‐mediated polarization of M2 microglia/macrophages	[27]
*Ginkgo biloba* extract	Decreasing microglial cell‐secreted inflammatory factor expression	[28]
12/15‐lipoxygenase (12/15‐LOX) inhibitor ML351	Repression of proinflammatory cytokines, together with the expression of the c‐Jun‐n‐terminal kinase	[29]
CXCR4 antagonist CX807	A reduction in glutamate‐mediated neuronal loss and microglial activation and anti‐inflammatory effects	[30]
17β‐estradiol	A reduced number of activated microglia or infiltrating monocyte‐derived macrophages selectively dampened the activation of the neuroinflammatory cascade	[31]
Administration of antithrombin (AT)	AT has an anticoagulant effect on macrophage/microglial activation and a direct anti‐inflammatory effect.	[32]
PPARγ agonist 15d‐PGJ2	Downregulation of TNF‐α and IL‐1 expression, decreasing apoptotic cells, and CD68 positive staining	[33]
Isosteviol Sodium (STV‐Na)	Regulation of microglia/macrophage polarization by the GAS5/miR‐146a‐5p sponge	[25]
Propofol	Inhibition of microglial hyperactivation via A2b receptors	[34]
Glatiramer acetate	Reducing infarct volume and proinflammatory mediators, promoting early neurogenesis, accelerating sensorimotor recovery, and preventing long‐term memory loss	[35]
HMG‐CoA reductase inhibitors	Preventing neuronal apoptosis and synaptic damage by blocking the NF‐κB signaling pathway to inactivate microglia	[36]
Gastrodin (GAS)	Decreasing cerebral infarct size, reversing blood–brain barrier damage, and reducing inflammation	[37]
Vx‐765	Shifting the polarization of microglia from the M1 phenotype to the M2 phenotype	[38]
Interleukin (IL)‐13	Enhancement of the ex vivo anti‐inflammatory response of microglia/macrophages and diminution of ischemia‐induced brain cell death	[39]
Montelukast	The phenotype of microglia is affected, improving fibrous reorganization and long‐term functional recovery after cerebral ischemia and promoting the recruitment and maturation of OPCs	[40]
α‐Lipoic acid	Inducing microglia to polarize toward the M2 phenotype and attenuating NF‐κB activation	[41]
CO‐releasing molecule‐3	Suppressing neuroinflammation and reducing blood–brain barrier damage	[42]
L‐3‐n butylphthalide	Shifting M1 microglia/macrophage polarization toward the M2 phenotype	[43]

Abbreviations: Akt, serine‐threonine protein kinase; BDNF, brain‐derived neurotrophic factor; miR, microRNA; mTOR, mammalian rapamycin; MCAO, middle cerebral artery occlusion; NF‐κB, nuclear factor‐κB; OGD, oxygen and sugar deprivation; STAT, signal transduction and transcriptional activator; TNF‐α, tumor necrosis factor‐α.

## 2 PATHOPHYSIOLOGY OF STROKE

A stroke is considered to be a sudden burst in the nervous system caused by damage to the perfusion of blood vessels to the brain, so an important step in studying neurovascular disease is to understand the anatomy of the nerve vessels. The brain's blood supply is mainly composed of two internal carotid and vertebral artery control when there is insufficient oxygen supply and support the brain can cause ischemic stroke, concerning reduced blood flow may induce the formation of thrombus, resulting in thrombosis cerebral apoplexy.^5^ Cerebrovascular obstruction caused by ischemia hypoxia for a long period of time, can lead to brain tissue necrosis, the arrival of necrosis, organelles swelling lipid membrane rupture cell contents into the intercellular space along with loss of neuronal function.^5^ Inflammation, blood–brain barrier (BBB) damage, oxidative stress cytokines, and leukocyte infiltration are all important factors in stroke pathology.^44^

## 3 OVERVIEW OF MICROGLIA

**Figure 2:**
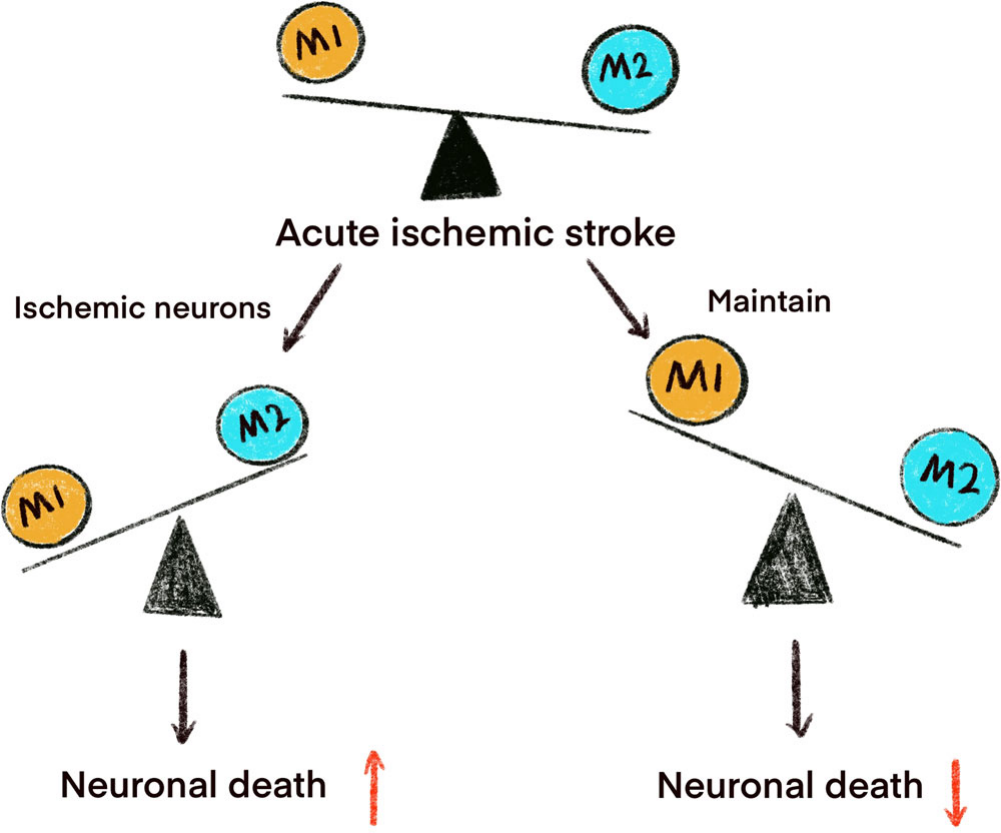
The roles of microglia in ischemic stroke. The balance between proinflammatory and anti‐inflammatory responses is important for determining outcomes after stroke. [Color figure can be viewed at wileyonlinelibrary.com]

Sierra et al.^45^ first proposed the term to describe glial cells and divided them into microglia, oligodendrocytes, and astrocytes. Microglia originate in embryonic yolk sacs and can be detected at 13 weeks of gestation in humans.^46^ Interleukin‐34, colony‐stimulating factor, and IFN regulatory factors have an impact on microglia progression.^47^ Microglia can protect the brain against the invasion of pathogens and play a key part in restoring brain function and disease recuperation. What's more, microglia can take on different forms under different conditions or circumstances. At standstill, microglia mainly take on the role of immunosurveillance and have the ability to eliminate cellular fragments and keep the central nervous system stable.^48^ Microglia are hypersensitive to external stimuli and can be activated to differentiate into M1 and M2, two types when minor pathological changes in the central nervous system occurs (infection, injury, ischemia, neurodegenerative disease, and disturbances in neuronal electrical activity).^49^ Classic activated M1‐type microglia can release proinflammatory mediators and lead to nerve damage,^50^ but selectively activated M2‐type microglia play a role in tissue repair, cell debris removal, and nutritional factor supply by secreting anti‐inflammatory mediators (Figure 2).^51^, ^52^, ^53^

## 4 RESEARCH PROGRESS OF MICROGLIA SIGNALING PATHWAY IN STROKE TREATMENT

**Figure 3:**
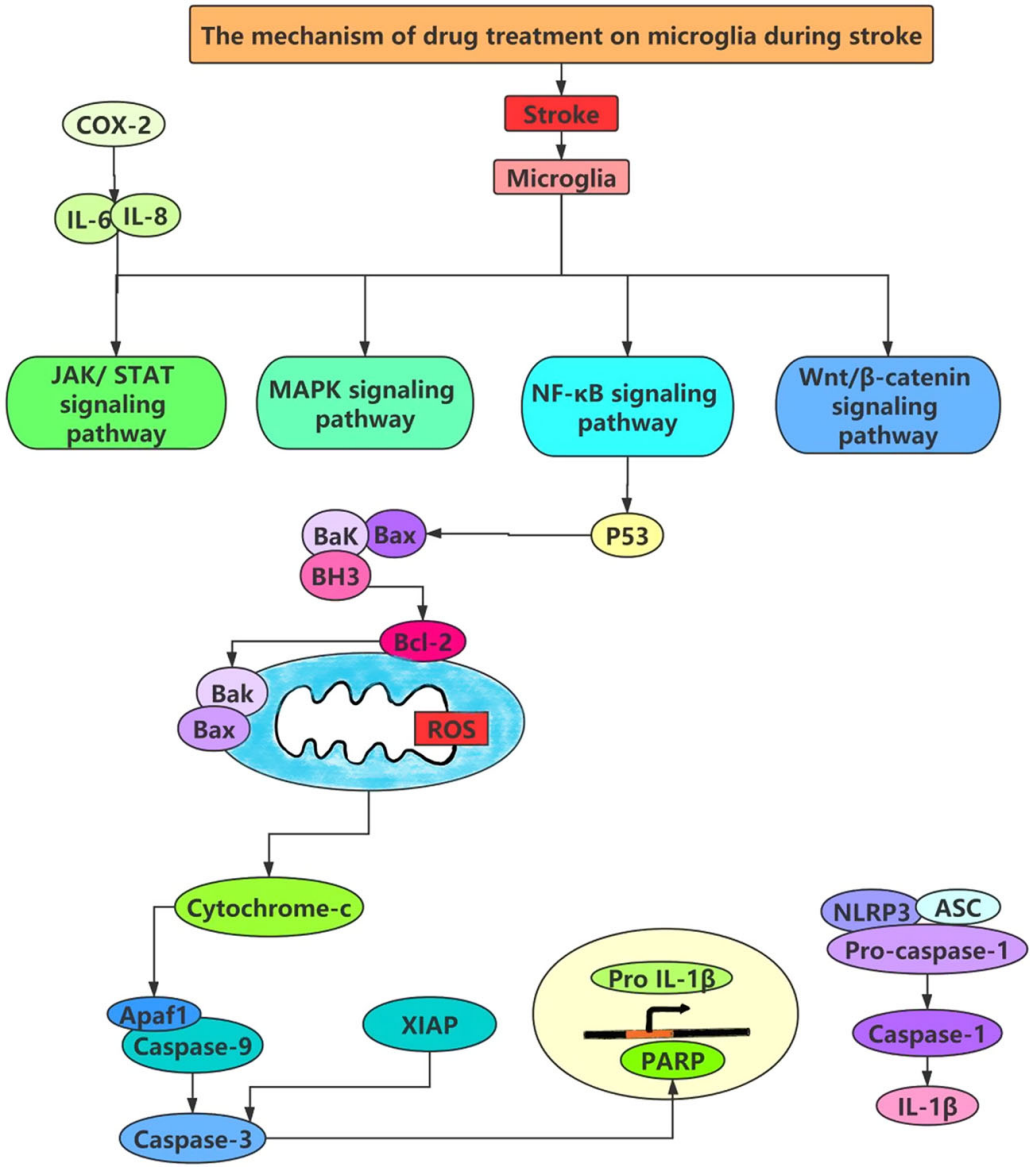
Microglia‐related signaling pathways in stroke treatment. AKT, serine/threonine‐protein kinases; AMPK, adenosine 5′‐monophosphate (AMP)‐activated protein kinase; IL, interleukin; JAK/STAT, Janus protein tyrosine kinase/signal transducer and activator of transcription; MAPK, mitogen‐activated protein kinase; NF‐κB, nuclear factor‐κ‐gene binding; ROS, reactive oxygen species. [Color figure can be viewed at wileyonlinelibrary.com]

Signaling pathways ischemia changes the microenvironment of microglia and activates microglia. Current studies emphasize that interference in intracellular signal regulation determines microglia status (Figure 3).^54^ The signaling pathways linked to ischemia‐induced microglial polarization are discussed in the next section (Table 2).

**Table 2:** Alternation of signaling pathways related to microglia phenotype after cerebral ischemia.

Signaling	Activity after ischemia	References
JAK/STAT	↓	[7, 22, 54, 55, 56, 57]
MAPK	↓	[11, 19, 55, 58, 59, 60]
NF‐κB	↓	[9, 10, 11, 12, 15, 36, 38, 55, 60, 61]
HDAC3‐p65‐cGAS‐STING	↓	[62]
Nrf‐2/Keap1	↓	[23]
AKT	↑	[11, 14]
OIP5‐AS1‐miR‐186‐5p‐CTRP3 axis	↑	[58]
IL‐4/STAT6/ST2	↑	[56]
BDNF/trkb	↑	[20]
AMPK	↑	[17, 24, 63]
p‐ERK	↑	[64]
Wnt‐3a	↑	[8]
Wnt/β‐catenin	↑	[13]
GPR120/b‐arrestin2	↑	[59]
GAS5/miR‐146a‐5p sponge	↑	[25]
PPARγ	↑	[26]
IL‐33/ST2	↑	[27]

Abbreviations: AKT, serine/threonine‐protein kinases; AMPK, adenosine 5′‐monophosphate (AMP)‐activated protein kinase; BDNF, brain‐derived neurotrophic factor; GAS, gastrodin; IL, interleukin; JAK/STAT, Janus protein tyrosine kinase/signal transducer and activator of transcription; MAPK, mitogen‐activated protein kinase; miR, microRNA; NF‐κB, nuclear factor‐κ‐gene binding; PPARγ, peroxisome proliferator‐activated receptor‐γ; p‐ERK, protein kinase R‐like ER kinase; STAT, signal transduction and transcriptional activator.

### 4.1 JAK/STAT signaling pathway

The JAK/STAT signaling pathway is implicated in many important biological processes, namely, cell proliferation, differentiation, apoptosis, and immune regulation. At the same time, a growing number of studies have demonstrated that the sustained activity of the AK/STAT signaling pathway is strongly associated with inflammatory diseases.^55^ Melatonin and xuesaitong altered the proinflammatory and anti‐inflammatory polarity of microglia while reducing neuronal cell death by reducing pSTAT3 (signaling sensors and activator of transcription 3) expression. They established a focal cerebral ischemia‐reperfusion (I/R) model in rats that were given gardenogiae extract GJ‐4 and nimodipine. Depressurization test, Morris water maze test, neurological function score, TTC (2,3,5‐Triphenyltetrazolium chloride) staining, Nissl staining, immunohistochemistry, Western blot, and other methods were used. In mechanism studies, GJ‐4 treatment has been found to inhibit Janus kinase 2 (JAK2)/signal transduction and transcriptional activator 1 (STAT1) pathway, thereby inhibiting microglia‐mediated neuroinflammatory responses.^6^ At the same time, Xiang et al.^65^ found that USP18 overexpression by lentivirus in mice with middle cerebral artery occlusion (MCAO) can inhibit microglial activation and inhibit the JAK/STAT pathway. Liu et al.^7^ and Li et al.^57^ in their studies on the mechanism of melatonin and xuesaitong in the treatment of stroke, respectively, proved that both drugs changed microglia from proinflammatory to anti‐inflammatory polarity, while at the same time reducing neuronal cell death by reducing pSTAT3 expression.^7^

### 4.2 MAPK signaling pathway

The four cascades of the extracellular signal‐associated kinase (ERK1/2), Jun amino‐terminal kinase (JNK1/2/3), p38‐MAPK, and ERK5 share the mitogen‐activated protein kinase (MAPK) signaling pathway, which is associated with cell proliferation, differentiation, migration, senescence, and apoptosis.^66^ Studies have shown that excessive salt intake can lead to proinflammatory M1 microglia polarization, worsening ischemic stroke, by a specific mechanism that promotes the expression of P38/MAPK upregulated AR proteins.^8^ Starossom et al.^67^ demonstrated that Galectin‐1 restrains microglia from producing inducible nitric oxide synthase (iNOS) and other inflammatory mediators mainly by activating the P38 MAPK signaling pathway, and it also increases CD45 phosphatase activity.

### 4.3 NF‐κB signal pathway

As a multifunctional nuclear transcription factor, NF‐κB is known to be present at several signaling pathways, and it occupies a very significant position in the inflammatory response. Mi et al.^9^ in lipopolysaccharide (LPS)‐induced inflammation of microglia cell line BV‐2, they found that a Ferula sinkiangensis K. M. Shen in Xinjiang inhibited NF‐κB signal pathway and then inhibited M1‐type polarization, ROS production, and NADPH oxidase activity. Cottonseed oil (CSO) significantly reduced microglia and astrocytes activation, causing a reduction of IL‐1β, IL‐6, and TNF‐α levels in rats with MCAO, which is mainly attributed to its inhibition of NF‐κB protein expression.^15^ Several researchers have found that paclitaxel has anti‐inflammatory and neuroprotective effects. They further discovered that paclitaxel achieves these functions predominantly by downregulating the expression of proinflammatory substances, such as iNOS production and messenger RNA (mRNA) expression of IL‐1β and that the mechanism underpinning this is the inhibition of NF‐κB signaling pathway and microglial activation.^10^ Other studies also shown that a number of drugs, which include mainly tanshinol borneol ester (DBZ), stepharine, BHDPC, and statins can prevent neuronal apoptosis and synaptic damage by suppressing NF‐κB activity to increase M2 mediator expression.^11^, ^12^, ^36^, ^68^

### 4.4 Wnt/β‐catenin signal pathway

In all animals, a variety of biological phenomena in developing and adult life is regulated by the WNT signaling cascade.^69^ Essential role of the Wnt/β‐catenin signaling pathway with regard to neural development, axon guidance, neuropathic pain relief, and neuronal survival.^70^ Wnt‐3a, a newly identified Wnt protein, can overactivated only the Wnt/β‐catenin signaling pathway, with no effect on other signaling pathways. In a study of Wnt‐3a, Zhang et al.^8^ revealed a significant decrease in CD68‐positive cells around brain infarcts, along with a decline in ionized calcium‐binding adapter molecule (Iba)‐1 protein levels. Besides, it also was a downregulation of nitric oxide enzyme and tumor necrosis factor expression. At the same time, Song et al.^13^ treated the mouse model of ischemic stroke with the Wnt/β‐catenin pathway activator TWS119 and found that TWS119 improves neuroinflammation in stroke by regulating the anti‐inflammatory phenotype of microglia.

### 4.5 Others

There are many other mechanisms involved in the use of drugs in the treatment of stroke. Related literature suggests that gingerol, a specific component in the daily consumption of ginger, resists the neuroinflammatory response triggered by microglia by blocking the Akt‐MTOR‐STAT3 pathway. Some researchers have observed that 6‐gingerol effectively inhibits the phosphorylation of serine‐threonine protein kinase (Akt), mammalian rapamycin, and STAT3 to achieve inhibition of microglia‐mediated neuroinflammation, and therefore conclude that 6‐gingerol can promote cerebral ischemia by inhibiting the Akt‐MTOR‐STAT3 pathway recovery from the injury.^14^ Liao et al.,^62^ using cell biology, molecular biology, and biochemistry, found that the proinflammatory microenvironment is formed through the activation of the microglial cGAS‐STING pathway by mitochondrial DNA and that HDAC3 was found to regulate P65 acetylation and nuclear localization in microglia to raise the expression of cGAS and augment the cGAS‐STING pathway activation. Microglia in the absence of cGAS or HDAC3 can block I/R‐induced neuroinflammation and brain injury, and this in vivo result further illustrates the essential role of the HDAC3‐P65‐cGAS‐STING pathway in the evolution of I/R‐induced neuroinflammation.^62^

Li et al.^71^ found that BHDPC had considerable neuroprotective effects on MCAO‐induced ischemic injury in mice, inhibited neuronal cell loss and apoptosis, attenuated BBB damage, and changes in tight junction proteins, thereby aiming to improve neurological deficits and cerebral infarction. In this study, BHDPC was also identified to diminish the activation of M1 microglia and conversely, it enhanced the polarization of M2, a function that can be diminished by knocking out genes in the PKA (protein kinase A)/CREB (CAMP response element‐binding protein) signaling pathway. In‐depth findings revealed that the role of BHDPC in the development of microglial cell inflammation was mainly associated with the inactivation of the NF‐κB signaling pathway and enhanced phosphorylation of PKA and CREB.^71^

## 5 ADVANCES IN MICROGLIA‐MEDIATED STROKE THERAPY

### 5.1 Inhibition of proinflammatory factor production

Chen et al.^58^ explored how 6‐curcumin improved neurological and reduced infarct size. It was to be found that levels of interleukin‐6, iNOS, and the proinflammatory cytokine interleukin‐1 β were markedly diminished in the 6‐curcumin‐treated group compared to the control group. In addition, proinflammatory cytokines secreted by microglia were inhibited by LPS‐stimulated microglia with 6‐gingerol.^14^

Neurological deficits, infarct volumes, neuronal damage, BBB damage, and brain edema can be alleviated by treatment with paclitaxel, CSO, and β‐theophylline (BCP), mainly due to a decline in IL‐1β, IL‐6, and TNF‐α levels.^8^, ^15^, ^16^ At the same time, it was also found in studies about baicalin that it restrained the performance of iNOS, TNF‐α, and IL‐1β expression in AM/BM.^17^

### 5.2 Toll‐like receptors

Pattern recognition receptors are an integral link between microglia and neurons to transmit information to each other^72^ Toll‐like receptors have been identified as critical pattern recognition receptors for pathogen‐associated molecular patterns and their ability to induce the synthesis of proinflammatory molecules through activation of NF‐κB factors for initiating proinflammatory immune responses.^73^ The literature indicates that primary microglia are triggered and then metamorphosed into M1 or M2 phenotypes via LPS, IFN‐γ, or IL‐4. Simultaneously, BCP was found to block microglial cell transformation and to decrease the production of proinflammatory factors. To be specific, BCP declined the number of activating M1‐type microglia and augmented the M2‐type microglia. The above results led to an improvement in the function of the nervous system. BCP diminished LPS + IFN‐γ‐induced proinflammatory cytokine secretion, downregulated the amount of TLR4 family proteins, and shifted microglia more toward the M2 phenotype. It was reported that the number of activated microglia and astrocytes was reduced in a model of arterial occlusion in the brain treated with CSO, and furthermore, that it inhibited the expression of TLR4.^15^ This gives an indication that the reduction in TLR4 activity mediates anti‐inflammatory action of BCP and blocks the switch to an M2 phenotype from M1 in microglia.^16^

Stepharine has an inhibitory effect on MCAO‐induced neurological dysfunction score, cerebral water content, and cerebral infarction volume in vivo. Stepharine inhibits lPS‐induced TLR4 expression. It confirms that Stepharine improves the prognosis of MCAO rats and reduces neuron loss by inhibiting the TLR4/NF‐κB pathway. Inhibition of overactivation of microglia.^12^ Ovariectomized female mice with middle cerebral artery occlusion were given the steroid hormones estrogen, G1, and ICI182780 after their replenishment. A new estrogen receptor G protein‐coupled receptor 30 (GPR30) was further expressed in primary microglias treated with oxygen and sugar deprivation (OGD) thus ischemic damage is more severe. Activation of GPR30 dramatically decreased the level of TNF‐α, IL‐1β, and IL‐6 in ischemic penumbra and microglia, simultaneously alleviating neuronal damage in OGD patients, as measured by CCK8 (cell counting kit‐8) and LDH (lactate dehydrogenase). Activation of GPR30 inhibited microglia cell activity. The rationale behind this manifestation is the diminution of TLR4 protein and Iba1 as well as the inhibitory activity of NF‐κB, hence the study demonstrates that TLR4‐mediated microglia inflammation can be inhibited by GPR30 to exert acute neuroprotective effects.^61^

### 5.3 NLRP3 inflammasome

Inflammasomes are multi‐protein complexes in which NLRP3 (NOD‐, LRR‐, and pyrin‐structural domain protein 3) can exacerbate the development of inflammatory diseases if overactivated.^74^ PAP1, a Kv1.3 channel blocker, was used to treat MCAO/R model mice. It was found that the number of NLRP3(+)/Iba1(+) cells, as well as the level of NLRP3 protein in vivo, decreased, as did the level of NLRP3 mRNA and protein expressed in vitro. Their study also discovered that the scores of neurological deficits in the MCAO/R model were improved and the infarct volume lessened.^18^ The NLRP3 inflammasome is primarily activated in microglia after cerebral I/R injury at first and is predominantly displayed in the neurons. Mitochondrial dysfunction exerts an essential role in the activation of the NLRP3 in microglia post‐OGD/R, a factor that is also inhibited by mitochondrial protectants in rats with brain I/R.^75^ It provides protection from ischemia‐induced nerve destruction and attenuates the microglia‐mediated neuroinflammation that occurs in the brain by reducing oxidative strain and spontaneous combustion.^76^

### 5.4 Antioxidant

Not only do antioxidants modulate neuroinflammation by suppressing the inflammatory gene expression but they also modify the intracellular antioxidant levels, thus favoring a shift in microglia phenotype from proinflammatory to anti‐inflammatory polarity and achieving neuronal protection. Liang et al.^76^ indicated that the accumulation of ROS could be reduced by inhibition of TREM‐1 after ischemia, reducing the content of malondialdehyde and other lipid peroxidation. Therefore, inhibition of TREM‐1 can lessen inflammation and burning death of MCAO rats. At the same time, studies have shown that Pterostilbene and Kellerin can decrease ROS production and decline nerve cell damage by inhibiting the NF‐κB signaling pathway.^9^, ^10^

In a study of his, 8E, a derived 3‐n‐butylphthalide which releases hydrogen sulfide (H2S), downregulated superoxide released from rat encephalic regions and primary cultured OGD microglia. Through using small interference RNA in combination with pharmacological inhibitors, it is further evidence that 8E regulated and activated microglia superoxide formation via the PI3Kγ/AKT/NOX2 signaling pathway so as to preventing the neuronal death of adjacent neurons.^19^

### 5.5 miRNA

MicroRNAs, as small RNAs that do not encode proteins, are involved in all biological processes while they are also an essential biomarker^77^ and have been used in studies of diseases, such as cancer, stroke, traumatic brain injury, and spinal cord injury.^78^, ^79^, ^80^, ^81^ Increasingly, studies are now moving away from considering them as just a biomarker and exploring in more depth the therapeutic potential of miRNA mimics for these diseases. miR‐98,^82^ miR‐363,^83^ miR‐122,^84^ miR‐378,^85^ and miR‐210^86^ were found to reduce infarct size and edema in animal stroke models, thereby alleviating ischemic injury. For instance, overexpression of miR‐98 in short‐lived middle cerebral artery occlusion (tMCAO) is accompanied by OGD models that decrease infarct size at post‐tMCAO in mice. miR‐98 diminished the infiltration of proinflammatory Ly6C(HI) white blood cells after stroke and decreased M1 microglia numbers from affected areas. To prove their conjecture, they showed how miR‐98 reduced the BBB penetration by shifting fluorescently labeled dextran in vivo and enhancing transendothelial resistance in vitro. To their surprise, they additionally discovered that there were significant improvements in dyskinesia after treatment with miR‐98.^82^

By selectively regulating OPA‐interaction protein 5 Antisense RNA 1 (OIP5‐AS1) and miR‐186‐5P, infarction volume, neuronal apoptosis, inflammation, and oxidative stress responses in MCAO/R rats were significantly increased. Meanwhile, the levels of OIP5‐AS1 and CTRP3 were downregulated, while the levels of miR‐186‐5P were upregulated. Functional studies have shown the central position of the OIP5‐AS1‐miR‐186‐5P‐CTRP3 axis that regulates microglia/macrophage activation and neuronal apoptosis. This was mainly reflected in the decrease of infarct volume and attenuation of oxidative stress by OIP5‐AS1 was upregulated.^58^

### 5.6 Others

Hsu et al.^20^ found that by regulating endogenous brain‐derived neurotrophic factor (BDNF) production, calycoyne isoflavone treatment could enhance the expression of BDNF/TrkB in the brain and make microglia cells change from active ameba state to static branch state, so as to improve ischemic stroke injury in mice. Their results demonstrated that nVNS enhanced neurological prognosis, reduced infarct volume, lifted M2 polarization of microglia, and marked a decrease of apoptotic neurons. In addition, nVNS can decline the expression level of the IL‐17A protein. Intranasal administration of RIL‐17A was found to result in the loss of nVNS‐induced M2 polarization in microglia, leading to a progressive diminution of the neuroprotective role of nVNS.^63^ In both ICH models, STAT6 was activated in microglia/macrophages. Intranasal delivery of IL‐4 nanoparticles can accelerate STAT6 activation and promote hematoma regression after intracranial hemorrhage. IL‐4 therapy improves long‐term functional recovery. ST2 KO (knockout) reduced the beneficial effect of IL‐4 after intracerebral hemorrhage. The construction of chimeric mice with bone marrow showed that STAT6 KO could worsen the prognosis of intracerebral hemorrhage in both the periphery and the central nervous system. STAT6 KO abated the ability of phagocytic cells to phagocytic brain tissue after intracerebral hemorrhage and primary cultured red blood cells. Transcription analysis showed that IL‐1 receptor‐like 1 (ST2) expression level in STAT6 KO mice microglia/macrophages decreased after intracerebral hemorrhage.^56^

After rhsTM was administered to mice, HMGB1 levels were dramatically diminished while M1 microglia in the infarct area were more active, resulting in a substantial increase in the survival rate of the mice. It can be concluded that rhsTM therapy is effective in improving neurological scores, motor coordination, survival, and preventing brain damage after stroke. In summary, TM improves functional prognosis by inhibiting HMGB1 upregulation and M1 microglia activation during delayed cerebral ischemia.^68^

The increase in the growth rate of CD11b(+)/CD45(low‐MED) microglia in the ischemic hemisphere can be restrained by the Na^+^/H^+^ exchanger (Nhe1) in CX3Cr1‐CRE(ER) and Nhe1(F/F) mice after stroke. However, it decreases the expression of CD86 and IL‐1 and diminishes the expression of GFAP(+) reactive astrocytes. No activated CD11b(+)/CD45(low‐MED) microglia or CD11b(+)/CD45(HI) macrophages were found in the mice with NHE1 neuronal deletion in the ischemic brain, but there was observed inhibition of the two cell activation in the white matter lesions. The definitive research demonstrated that Nhe1 in microglia can elicit a proinflammatory response and therefore participate in the brain tissue repair process after stroke, yet Nhe1 in neuronal cells directly mediates acute ischemic neuronal injury, without any association with inflammation.^87^

In an article by Ren et al.,^59^ it was shown that dodecahexaenoic acid (DHA) effectively controls OGD‐induced inflammatory responses in microglia and mouse microglia BV2. The mechanism of this phenomenon is due to DHA can dampen OGD‐induced inflammation through the interplay between G protein‐coupled receptor 120 (GPR120) and β‐Arrestin2. At the same time, inhibiting GPR120 could remove the inhibitory action of DHA, which leads to more intense OGD‐induced inflammation.^59^

Gan et al.^88^ demonstrated that CD21 dose‐dependence improved neurological function in tMCAO mice with transient middle cerebral artery occlusion. Biochemical analyses demonstrated that a major role of CD21 was seen in the reduction of M1 expression and the increase of M2 expression. Further studies have shown that CD21 is primarily characterized by reducing the production of inflammatory factors and promoting the production of anti‐inflammatory factors. More importantly, the activity of phosphorylated adenosine 5ʹ‐monophosphate‐activated protein kinase (AMPK) could be increased by CD21 and they also found that AMPK inhibitor (compound C) could flip the role of CD21 in BV2 cells. The above findings suggest that CD21 may achieve induction of M2 polarization in microglia through activation of AMPK, thereby reducing the neuroinflammatory response after ischemia.^88^

Quantitative stereology, immunofluorescence microscopy, and flow cytometry were used in vitro. Using microglia co‐localization and proliferative markers, they noticed multiple growths in one location of microglia as well as proliferating microglia within the preconditioning semicortex 72 h after ischemic preconditioning (IPC). At the same time, microglia changed shape and increased body volume, indicating phenotypic activation. They used transgenic mouse models of bifurcated protein receptor (CX3CR1) ensemble or systemic type I IFN signaling deficiency to determine that microglia proliferation after IPC is dependent on bifurcated protein signaling, but not type I IFN signaling.^89^

Eerdun Wurile (EW) was used to treat a rat model of MCAO. Diminished neurological deficits treated with EW (*p* < 0.01), but cerebral blood circulation was superior in the lesioned group compared to the control group, and 186 genes were upregulated in expression. Among them, IGF1 (*p* < 0.01) was upwardly revised after EW treatment. Meanwhile, it dramatically enhanced the expression of secreted proteases, complementary components, and microglial cell markers. These findings suggest that EW promotes the production of neurons and microglia growth factors IGF1 and IGF2.^21^

## 6 QUESTIONS AND PROSPECT

**Figure 4:**
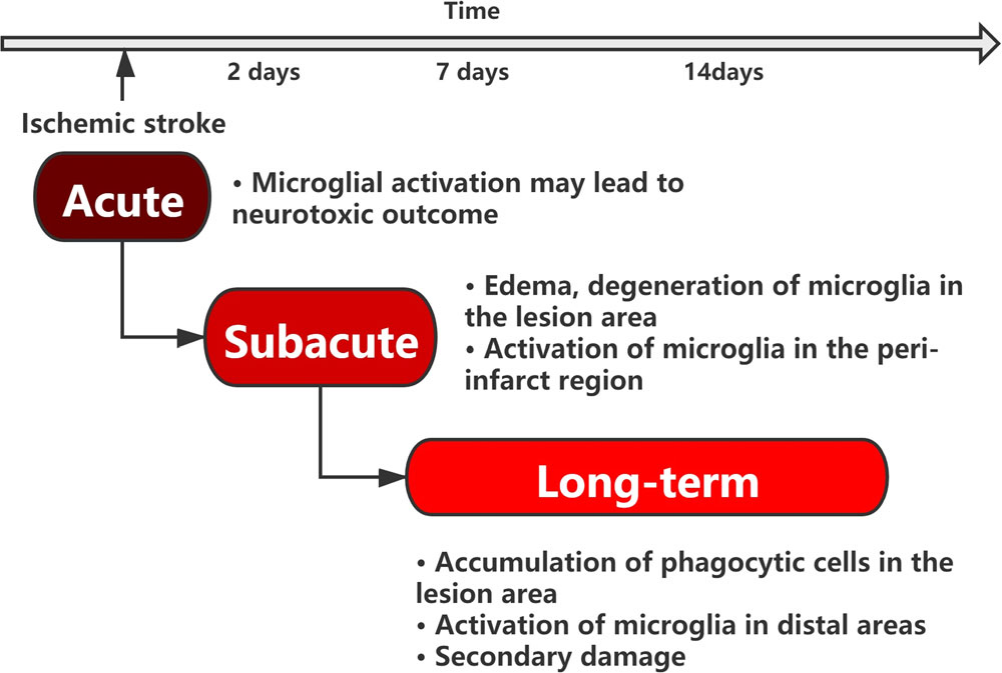
Time‐scale of microglia‐mediated events after ischemic stroke [Color figure can be viewed at wileyonlinelibrary.com]

As research into stroke and microglia has progressed, it has become apparent that the polarization of microglia has a dramatic impact on the development of stroke. Early stroke leads to an inflammatory response that facilitates recovery, but if left untreated the inflammatory response can exacerbate the damage to brain tissue when the body is left in a state of prolonged ischemia (Figure 4). The majority of these drugs have been found to work by promoting the conversion between the M1 and M2 phenotypes of microglia to inhibit the inflammatory response and, in a few cases, by directly inhibiting microglial activation, resulting in the protection of brain tissue. The changes between M1 and M2 phenotypes of microglia have a key role in the regulation of brain tissue injury repair; therefore, in future studies, we need to shift the focus of our research from inhibiting microglia activation to finding an optimal balance between inhibiting early M1 phenotype‐polarized microglia and promoting dynamic changes in late M2 phenotype‐polarized microglia. This allows the natural process of hematoma clearance and brain repair to be maximized. However, in many experiments, the majority of ischemic stroke models are short‐term ischemic, but in practice, most stroke patients are not treated on time. There are serious questions as to whether these results can be used in clinical practice, which requires the development of animal models of cerebral hemorrhage that are more similar to clinical disease. Second, the experimental animal models did not take into account the effect of gender on the results, so future studies could increase the gender grouping to facilitate the translation of the results to the clinic. Future research should focus on the specific regulatory mechanisms of M1/M2 polarization in microglia, providing new entry points for drug interventions in the treatment of cerebral hemorrhage and new directions for the treatment of cerebral hemorrhage diseases.

## AUTHOR CONTRIBUTIONS

Ling‐Jing Zhang, Hong‐Su Zhou, Shi‐Ya Wang, Yi‐Huan Guan, Chao Zhang, and De‐Rong Fang completed the manuscript. All authors have read and approved the final submitted manuscript.

## FUNDING

Funding information is not applicable to this article.

## CONFLICTS OF INTEREST

The authors declare no conflicts of interest.

## TRANSPARENCY STATEMENT

The authors affirm that this manuscript is an honest, accurate, and transparent account of the study being reported.

## ETHICS STATEMENT

The ethics statement is not applicable to this article.

## Supporting information

Supporting information.

## Data Availability

Data sharing is not applicable to this article as no data sets were generated or analyzed during the current study.
