# Supplementary material for: Changes in microglia during drug treatment of stroke
Source: Ibrain. 2022 May 22;8(2):227–40. doi: 10.1002/ibra.12037 (PMC10528798; doi:10.1002/ibra.12037)

**Copyright Transfer Statement**

Copyright Transfer Statement

Each author must read and sign the following statement.

Completed second page of this form should be submitted to the Editorial Office:

No 88, Keyuan South Road, Tianfu Life Science Park；Chengdu, China 610041.

E-mail: Ibrain_editor@idragon.org.cn; Tel: 028-85164141.

TRANSFER OF COPYRIGHT

All articles published in this journal are protected by copyright, which covers the exclusive rights to reproduce and distribute the article (e.g., as offprints), as well as all translation rights. No material published in this journal may be reproduced photographically or stored on microfilm, in electronic data bases, video disks, etc., without first obtaining written permission from the publisher . The use of general descriptive names, trade names, trademarks, etc., in this publication, even if not specifically identified, does not imply that these names are not protected by the relevant laws and regulations.

Transfer of copyright to becomes effective if and when a Copyright Transfer Statement is signed or transferred electronically by the corresponding author. The copyright to this article, including any graphic elements therein (e.g. illustrations, charts, moving images), is assigned for good and valuable consideration to Ibrain effective if and when the article is accepted for publication and to the extent assignable if assignability is restricted for by applicable law or regulations.

Each author certifies that he or she has No business cooperation (e.g., equity interest, patent/licensing arrangements, etc.) that might pose a conflict of interest in connection with the submitted article. All funding sources supporting the Work and all institutional or corporate affiliations of the authors are acknowledged in a footnote in the Work. All experimentation was conducted in conformity with ethical and humane principles of research.

**Copyright Transfer Statement**

JOURNAL TITLE: Ibrain MANUSCRIPT TITle:The mechanism of drug treatment on microglia during stroke

We are in agreement with the statements mentioned in TRANSFER OF COPYRIGHT of Ibrain the above-mentioned requirements. We accept scientific

and legal responsibility of the article.

| **Surname and Name (in**  **capital letters)** | **contribution** | **Signature** |
| --- | --- | --- |
| **Ling-Jing Zhang** | completed the manuscript | *Ling-Jing Zhang* |
| **Hong-Su Zhou** | completed the manuscript | *Hong-Su Zhou* |
| **Don-Qin Chen** | completed the manuscript | *Don-Qin Chen* |
| **Shi-Ya Wang** | completed the manuscript | *Shi-Ya Wang* |
| **Yi-Huan Guan** | completed the manuscript | *Yi-Huan Guan* |
| **Ya-Dan Deng** | completed the manuscript | *Ya-Dan Deng* |
| **Liu-Lin Xiong** | designed and polished the paper. | *Liu-Lin Xiong* |

........................................................................................................................(and more)

**Corresponding Author (Signature): Liu-Lin Xiong**

**Date: 2021.08.19**

**Mailing Address and Telephone/Fax Numbers:** 2633239421@qq.com
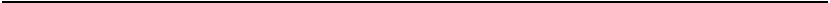

Supplement: Supplementary file 1 — Supporting information. [file IBRA-8-227-s001.doc]
